# Utility of a novel integrated deep convolutional neural network for the segmentation of hip joint from computed tomography images in the preoperative planning of total hip arthroplasty

**DOI:** 10.1186/s13018-022-02932-w

**Published:** 2022-03-15

**Authors:** Dong Wu, Xin Zhi, Xingyu Liu, Yiling Zhang, Wei Chai

**Affiliations:** 1 Senior Department of Orthopedics, The Fourth Medical Center of PLA General Hospital, Beijing, China; 2 National Clinical Research Center for Orthopedics, Sports Medicine and Rehabilitation, General Hospital of Chinese PLA, Beijing, China; 3 Longwood Valley Medical Technology Co. Ltd, Beijing, China; 4 School of Life Sciences, Tsinghua University, Beijing, China; 5 Institute of Biomedical and Health Engineering (iBHE), Tsinghua Shenzhen International Graduate School, Beijing, China

**Keywords:** Computed tomography, Deep neural network, Image segmentation, Hip joint, 2D U-Net

## Abstract

**Purpose:**

Preoperative three-dimensional planning is important for total hip arthroplasty. To simulate the placement of joint implants on computed tomography (CT), pelvis and femur must be segmented. Accurate and rapid segmentation of the hip joint is challenging. This study aimed to develop a novel deep learning network, named Changmugu Net (CMG Net), which could achieve accurate segmentation of the femur and pelvis.

**Methods:**

The overall deep neural network architecture of CMG Net employed three interrelated modules. CMG Net included the 2D U-net to separate the bony and soft tissues. The modular hierarchy method was used for the main femur segmentation to achieve better performance. A layer classifier was adopted to localise femur layers among a series of CT scan images. The first module was a modified 2D U-net, which separated bony and soft tissues; it provided intermediate supervision for the main femur segmentation. The second module was the main femur segmentation, which was used to distinguish the femur from the acetabulum. The third module was the layer classifier, which served as a post-processor for the second module.

**Results:**

There was a much greater overlap in accuracy results with the “gold standard” segmentation than with competing networks. The dice overlap coefficient was 93.55% ± 5.57%; the mean surface distance was 1.34 ± 0.24 mm, and the Hausdorff distance was 4.19 ± 1.04 mm in the normal and diseased hips, which indicated greater accuracy than the other four competing networks. Moreover, the mean segmentation time of CMG Net was 25.87 ± 2.73 s, which was shorter than the times of the other four networks.

**Conclusions:**

The prominent segmentation accuracy and run-time of CMG Net suggest that it is a reliable method for clinicians to observe anatomical structures of the hip joints, even in severely diseased cases.

## Introduction

Total hip arthroplasty is the most effective treatment for severe hip osteoarthrosis [1–3]. Preoperative computed tomography (CT)-based 3D planning is essential for total hip arthroplasty. Precise localisation and segmentation of the hip joint on CT images are necessary to simulate the placement of joint implants [4, 5]. CT images from diseased hips exhibit image degradation, noise, non-homogeneous intensities and obscure boundaries between the femoral head and acetabulum; because of these features, automatic CT hip-joint segmentation is challenging [6, 7]. Therefore, a computer-aided segmentation scheme is necessary for the fully automated segmentation of hip joints [8].

Various methods have been proposed to solve these problems (Table 1) [4, 9, 10]. Deep learning methods, particularly deep convolutional neural network (CNN)-based methods (e.g. V-Net and U-Net), have been successfully applied in hip segmentation [11]. Wang et al. [12] segmented pelvises using both 2D U-Net and 3D U-Net; they found that the dice overlap coefficient (DOC) was > 94%. Chu et al. [13] automatically segmented hip CT images using Spring SpringMvc MyBatis-based methods; the DOC reached 95%. Despite improved segmentation quality, these deep neural networks are time-consuming and demand a large amount of pixel-labelling. Moreover, these networks cannot manage holes and noise in segment results. Therefore, improvements are needed in segmentation using deep learning networks. This study aimed to develop a novel deep learning network, namely CMG Net, that could achieve accurate segmentation of femurs and pelvises.

**Table 1:** Solutions for segmentation of CT images in previous research

	Year of publication	Title of article	Contribution	Advantage	Inconvenient
1	2017	Accurate pelvis and femur segmentation in Hip CT with a novel patch-based refinement	They have presented a coarse-to-fine hip CT segmentation framework that consisted of region growing-based preprocessing, CRF-based coarse segmentation and patch-based refinement. The experimental results on 60 CT hips (120 hemi-hips) demonstrate the effectiveness of their method	(1) It starts with coarse segmentation techniques for obtaining the bone boundaries, followed by the bone refinement using a patch-based algorithm that is navigated by the extracted bone boundaries (2) GLCM is first used for bone classification and its effectiveness is demonstrated (3) the existing methods perform the label propagation for all voxels of the image to be segmented	(1) The total hip must be included in each volume. The position normalization for VOI is based on whole hip, so it will be a wrong indication in partial hip CT data (2) The highly refined manual segmentation is required. For each training sample, the radiology experts put a significant amount of effort into completing it (3) The method needs a long time computation
2	2018	Automated muscle segmentation from CT images of the hip and thigh using a hierarchical multi-atlas method	In this paper, they proposed a hierarchization of the multi-atlas method to reduce the inter-patient variability in muscles. Intermediate segmentation results of more easily segmentable structures, that is, skin, bones, and entire muscle, were effectively utilized for spatial normalization to reduce inter-patient variabilities of the final target structures of individual muscles	Significant improvement was observed by using the proposed hierarchical strategy. Although the individual muscles have large inter-patient variabilities, the spatial normalization using the region pre-segmented in the previous stage reduces the inter-patient variability. Significant improvements in accuracy were observed among all individual muscles around the thigh segment	A limitation of the proposed method, especially in its application in biomechanical simulation, is the lack of imaging of tendons, ligaments, and attachment regions
3	2017	Fully automated segmentation of a hip joint using the patient-specific optimal thresholding and watershed algorithm	This study proposed a fully automated segmentation method for a hip joint using the complementary characteristics between the patient-specific optimal thresholding and the watershed algorithm	The thresholding method generates patches which are often not closed but contain regional information; and the watershed algorithm generates patches which always have closed boundaries but have no regional information Clinical case studies with eight sets of CT scan data demonstrated that the proposed method can reliably segment a hip joint with high speed and accuracy without the aid of a prerequisite dataset and user manual intervention	(1) The proposed method was validated only with eight cases. (2) The accuracy of the proposed method is affected by the closed patches of the watershed algorithm (3) A use of primitive spheres in the proposed method may not be effective in the CT scan data where the femoral head is severely deformed due to diseases such as avascular necrosis

## Materials and methods

### Materials

This study was approved by the ethics committee of the General Hospital of People’s Liberation Army (IRB number: S2019-052-01). Demographic data are presented in Table 1. CT images were acquired using the Phillip CT Brilliance ICT with 1.00-mm slice thickness and 512 × 512 image resolution. The images were stored as unsigned 12-bit integers from 0 to 4095. For manual labelling of the hip joints, all images were automatically segmented using the thresholding technique, with a threshold of 200 Hounsfield units, on an in-house software (Mimics Research 19.0). Two experts then manually inspected the non-segmented areas of the femur and acetabulum. Slice-by-slice manual segmentation was used as the benchmark for the evaluation of distinct CNN structures. The number of slices per CT ranged between 200 and 600. Because the CT scans had different numbers of slices, the mean segmentation times per CT image were evaluated, rather than the mean segmentation times of the whole CT dataset.

### Datasets

To validate our proposed method for hip joint segmentation, we established a CT dataset consisting of 100 normal hips for segmentation (training subset: 70; test subset: 30). The osteoarthritis (OA) hip-joint segmentation dataset consisted of 100 CT images training set: 70; test set: 30); the developmental dysplasia of the hip (DDH) hip joint segmentation dataset consisted of 138 CT images (training set: 70; test set: 68); the femoral neck fracture (FNF) hip joint segmentation dataset consisted of 366 CT images (training set: 243; test set: 123); and the osteonecrosis of femoral head (ONFH) hip joint segmentation dataset consisted of 111 CT images (training set: 50; test set: 61). An overview of the datasets is presented in Table 2. Cases with metal components were excluded because of the potential influence of artefacts.

**Table 2:** Demographic information of patients enrolled in this study

Characteristic	FNF	ONFH	DDH	OA	Normal
Gender (M/F)					
Male	127	78	58	56	65
Female	239	33	80	44	35
Age	71.08 ± 15.24	50.32 ± 14.15	50.36 ± 14.04	57.74 ± 11.99	52.74 ± 13.4
Stage					
I	22	5	33	NA	NA
II	33	9	58	NA	NA
III	213	22	23	NA	NA
IV	98	75	24	NA	NA

^a^FNF was classified by Garden classification criteria. ONFH was classified by ARCO classification criteria. DDH was classified by Crowe classification criteria

### Establishment of network architecture

**Fig. 1:**
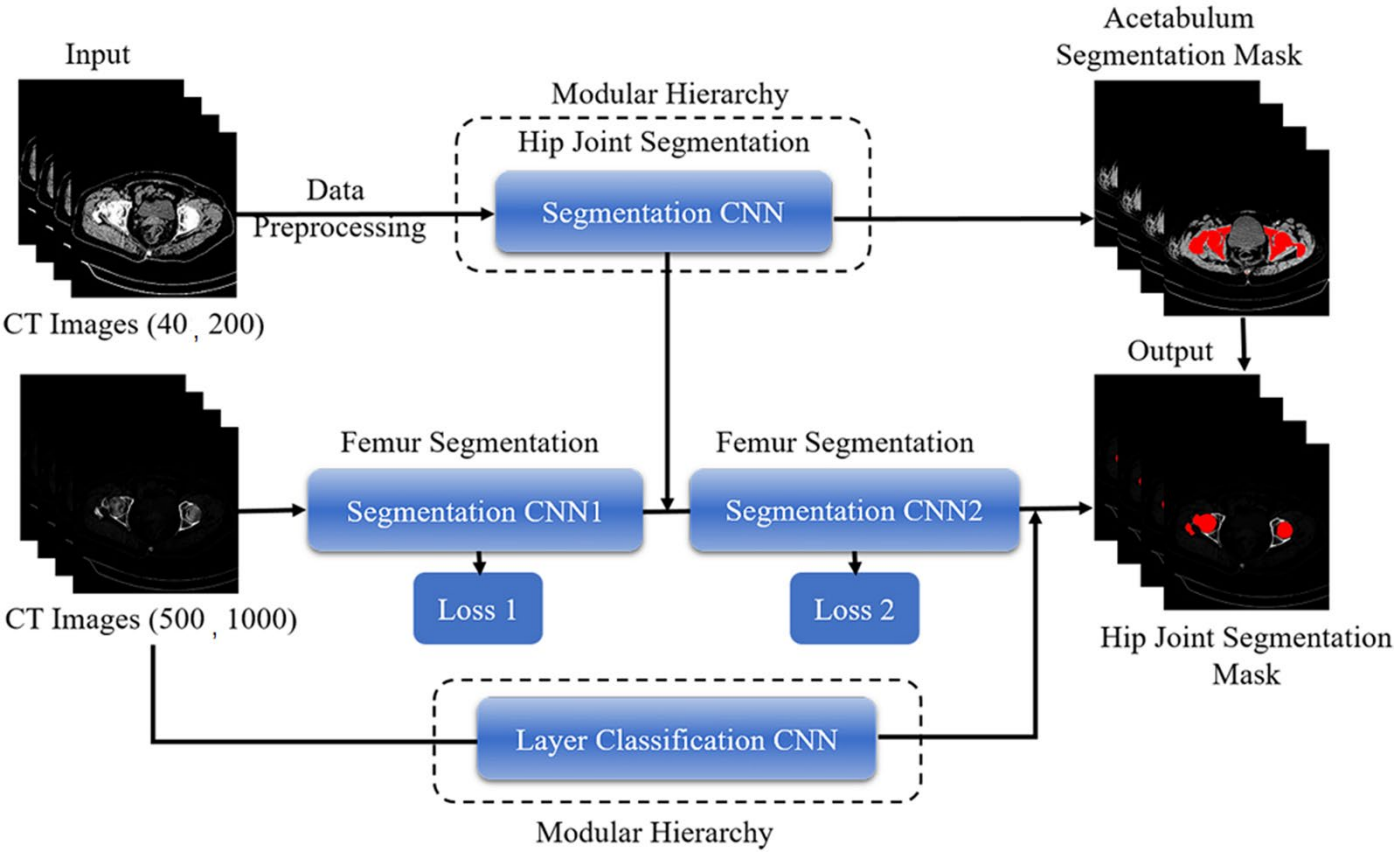
The flowchart of our proposed segmentation method. The input CT to the upward network has window level 40, window width 200 to highlight all bone structure. And the CT input to femur segmentation network has window level 500, window width 1000 to remove soft tissues and preserve bone structure as much as possible

We proposed a new network structure for femur segmentation in industrial use. The entire network was constructed in a modular hierarchy structure (Fig. 1). There were two main advantages. First, we embedded dense connections in a stacked hourglass segmentation network, which could accelerate the learning progress using fewer parameters. The number of stacked layers could be adjusted for tasks with different complexities. Second, we appended several different functional modules in the hierarchical structure for intermediate supervision to enhance the accuracy of different modules. They could be trained independently and followed by integration, facilitating future maintenance and model updates.

In our network, we adopted multiple new techniques to ensure the performance of femur segmentation on CT layer scans. We constructed the entire network in a modular hierarchy structure [14], comprising upstream, midstream, and downstream layers. The upward net was a modified 2D U-net that separated bone and soft tissues; it retained only the bony areas. The modified U-net had 2 × 2 max-pooling layers and 2 up-sample deconvolution layers, with additional 3 × 3 convolution layers included.

The bone and soft tissues were separated using the upward 2D U-net. The middle segmentation net acquired the main femur structures, while the downward net was a layer classifier to localise femur layers among multiple CT scan images. We trained these nets independently during the training process. In particular, we fed benchmark labels of the upward and downward nets to the main femur segmentation net in the training process then assembled the nets during the testing process.

The upper net acquired the feature map of the bone structures via separation of soft tissue and bones; it then fused the acquired map into the main network to increase the accuracy of bone segmentation. The loss function of the upward segmentation network is the combination of dice loss and softmax cross-entropy loss, represented as $L=a\cdot diceLoss+b\cdot crossEnropy$, where *a* and *b* are hyper-parameters. Dice loss could benefit the overall shape integrity; pixel-wise soft-max classification could benefit and preserve pixels in the bone edge area.

**Fig. 2:**
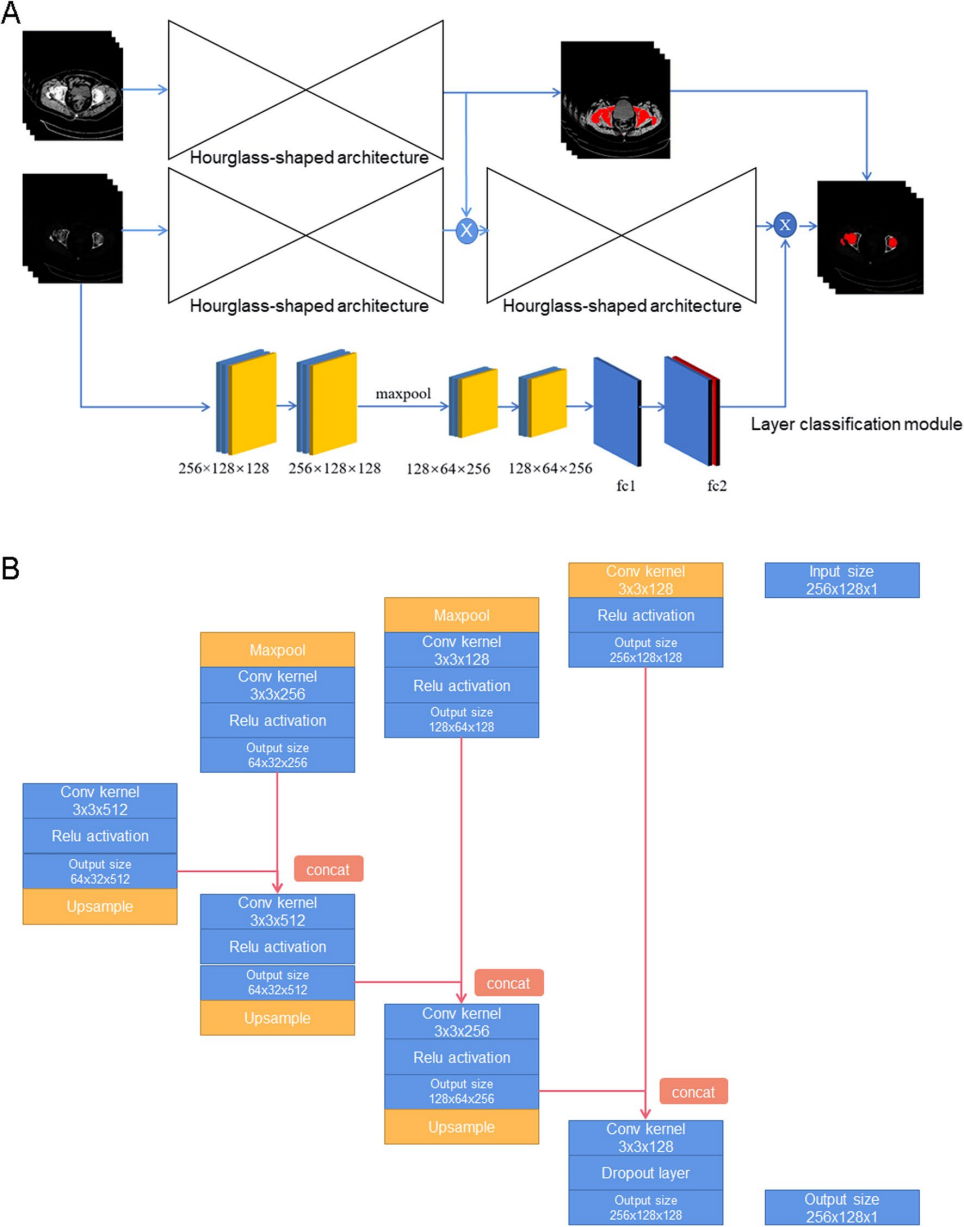
Establishment of network architecture. **A** A schematic view of the overall deep neural network architecture for the automatic segmentation of the hip joint. **B** The detailed parameters of the hourglass-shaped architecture

The middle net was the main segmentation net that constructed the femur, while the downward net was a layer classifier to localise femur layers among multiple CT scan images, most of which did not include femurs. We trained upward, middle, and lower nets together during the training process. In particular, we fed the feature map of the upward net to the main femur segmentation net during the training process to facilitate femur segmentation net focus on bony areas. Importantly, the network structure was composed of three modified 2D-U net models and a classification model. Each Unet model was composed of basic convolution, pooling, and up-sample deconvolutions. Because the pooling layer does not participate in the backpropagation calculation, it was not included in the calculation. Each Unet model had 18 layers, while the classification model had 14 layers. As an alternative, the lower net could be trained separately; thus, the structure enabled the inclusion of more functions in the neural networks without the loss of flexibility, and combinations of the results of the three nets were expected to increase the accuracy (Fig. 2A).

For the main femur segmentation network, the fundamental structure was an encoder–decoder with two 2 × 2 pooling layers and two up-sample deconvolutions. We stacked two encoder–decoder structures and densely concatenated all corresponding layers, thus combining the advantages of U-Net and Dense-Net [15]. The network could focus on information of different scales with the stacked U-net structure; the gradient could attenuate from back to front without disappearing or exploding because of the dense connection structure. Dense connections required fewer parameters and layers to achieve better performance, which is important for commercial purposes. In addition, we combined the two losses of both decoders’ output nodes, using the same loss of the upward network; we tuned the weights of the losses using the intermediate supervision to largely avoid a vanishing gradient and accelerate the learning progress.

The input image size was 256 × 256. We conducted data augmentation to cut the image in the middle and then flip the right part such that it appeared to be the “left” part. This yielded two 256 × 128 images from one 256 × 256 image. We performed this augmentation for both the training and testing processes. Detailed parameters of the hourglass-shaped architecture are shown in Fig. 2B.

For the downward layer classification net, we mainly used the network for two purposes. First, we located femur layers quickly and precisely among the CT scans (generally, only a few dozen CT layers have femurs among hundreds of CT scans; thus, feeding the entire data into the main segmentation network could decrease efficiency). Second, we provided additional classification confidence as a coefficient for the femur segmentation map to remove false segments; if a CT scan is not likely to have femurs in it, then the confidence of all positive segmentation results should be reduced.

### Model performance evaluation and statistical analysis

Segmentation performances of these CNN structures in different CT images were evaluated using DOCs and the Hausdorff Distance (HD). We defined the automatically segmented set of voxels as AS and the manually defined ground truth as GT [16].

1. The DOC quantified the match between two sets by normalising the size of their intersection over the mean of their sizes, defined as follows:
  $${\text{DOC}} = \frac{{2\left| {AS \cap GT} \right|}}{{\left| {AS} \right| + \left| {GT} \right|}}$$
  where the operator |·| returns the number of voxels contained in a region.
2. Distance-based metrics

Before the establishment of distance-based metrics, we defined a distance measure for the voxel “*x*” from a set of voxels “*A*” as:

$$d\left( {x,A} \right) = \mathop {\min }\limits_{y \in A} d\left( {x,y} \right)$$

where *d*(*x*, *y*) is the Euclidean distance of the voxels incorporating the real spatial resolution of the volume data.

We then defined the directed Hausdorff measure as the maximum distance between the point set *A* and the point set *B* for all points in *A* to the closest point in *B*. Mathematically, this was represented by the following equation:

$$\overrightarrow {{d_{H} }} \left( {A,B} \right) = \mathop {\max }\limits_{x \in A} \left( {\mathop {\min }\limits_{y \in B} \left( {d\left( {x,y} \right)} \right)} \right)$$

Thus, HD was defined as the maximum distance between two objects:

$$HD = \max \{ \overrightarrow {{d_{H} }} \left( {A,B} \right),\overrightarrow {{d_{H} }} \left( {B,A} \right)\}$$

We compared our proposed segmentation method with four CNN-based methods: fully convolutional network, 2D U-Net, 2.5D U-Net, and 3D U-Net (all popular methods for medical image segmentation). The comparison was composed of three parts. The first part compared the learning curves of the five nets by validating the loss in the training process. The second part tested the performances of CMG Net and the other nets in the segmentation of normal hip joints. We used the training set of normal hip joints to train each net and then used the test set of normal hip joints to validate the performances of the nets. The third part tested the performances of CMG Net and the other nets in the segmentation of diseased hip joints, particularly joints with severe disease. We used the training sets of FNF, ONFH, DDH, and OA to train each net separately and then used the test sets of each disease to validate the performance of each net. The training and test sets belonged to the same disease. We compared the times that those nets consumed and the parameters mentioned above using paired *t* tests and multiple comparisons in the general linear model. All analyses were performed using SPSS Statistics software, version 23 (IBM Corp., Armonk, NY, USA); *p* values < 0.05 were considered statistically significant.

### Training details

We use the Tensorflow 1.15 and NVIDIA RTX 2070 to train the network, which required 6 h, 30,000 iterations, and 10 epochs.

## Results

### CMG Net is effective for the segmentation of normal hip joints

**Fig. 3:**
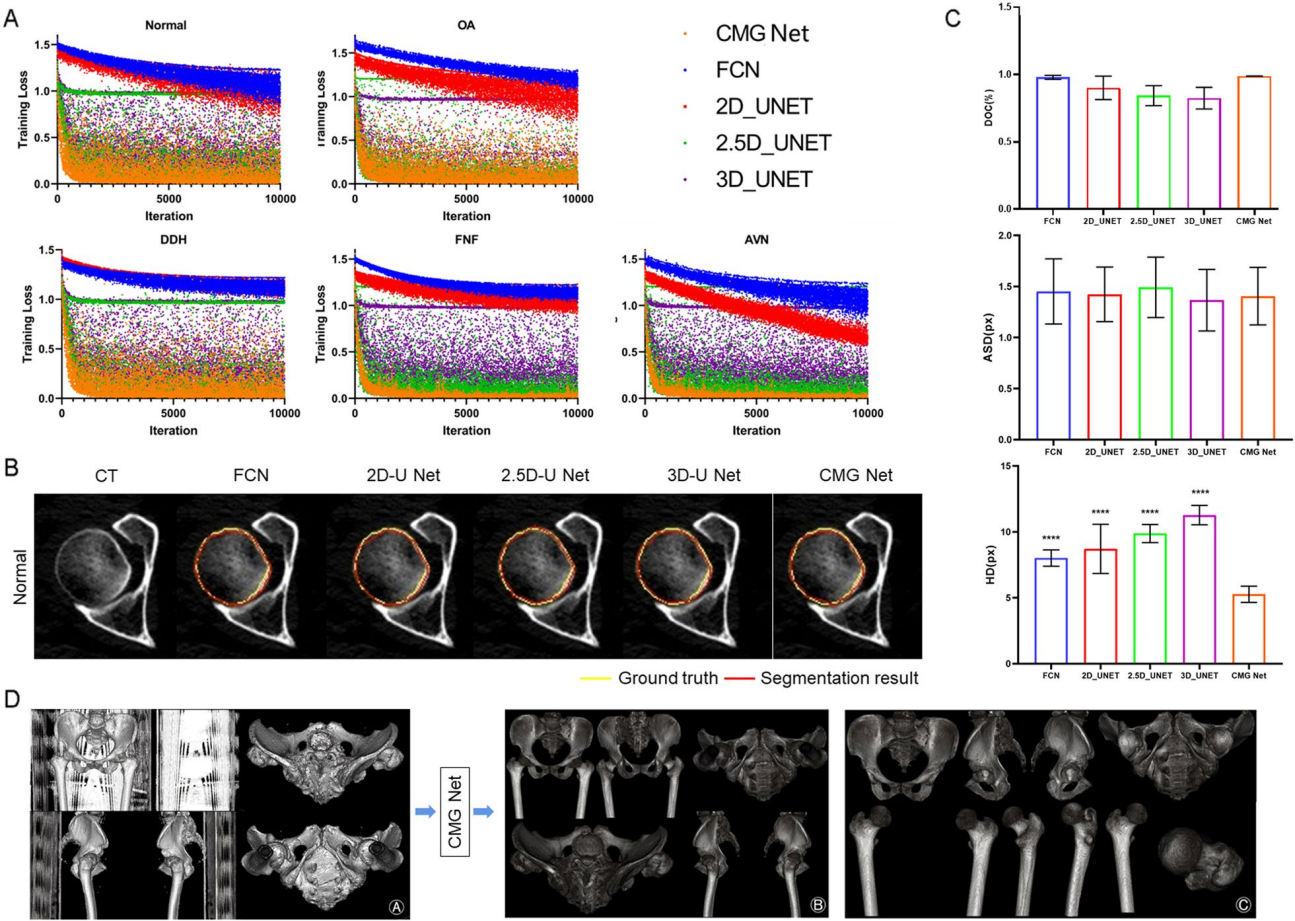
CMG Net is effective for segmentation of normal hip joints. **A** Comparison of learning curves from the training data with the proposed CMG Net and other alternative Net (Since we tune the class weights for different networks to ensure its performance so they don’t have to converge to one loss during training process.) **B** Qualitative comparison of the segmentation results obtained by the automatic segmentation to manual segmentation on a given axial slice of the normal hip joint. **C** Quantitative comparison of the segmentation results obtained by the automatic segmentation to manual segmentation on the normal hip joint. **D** After we piled up all the segmented layers according to the original CT sequence of a normal hip, we could rebuild an accurately segmented 3D hip model. **A** 3D model rebuilt by original CT images; **B** 3D model rebuilt by CT images segmented by CMG Net; **C** the anatomical sturctures of both femur and acetabular can be observed clearly. ***p < 0.001, ****p < 0.0001 versus CMG Net

The demographic data of all patients are shown in Table 2. This technique can be used in preoperative planning for total hip arthroplasty [17]. However, the use of this technique is limited to patients without metal implants because metal artefacts could influence the segmentation process. The training loss of the learning curves consistently decreased, demonstrating that there was no serious over-fitting (Fig. 3A). Comparison analyses indicated that CMG Net converged much faster than did the other four nets, particularly during the early learning stage. These results demonstrated that the proposed CMG Net could effectively accelerate the training procedure by overcoming optimisation difficulties via management of training in all upper, middle, and downward layers in the network.

Next, we used the test set of normal hip joints to evaluate the segmentation accuracy. Manually annotated boundaries were used for the benchmark. The consistencies between the boundaries of the acetabulum and femoral head were effectively labelled in most cases (Fig. 3B). Moreover, we used DOC, ASD, and HD to quantify the segmentation performance (Fig. 3C). The mean DOC of CMG Net was 98.99% ± 0.14%, which exceeded the performances of the other four nets. In addition, HD was computed as the mean longest distances from the surface model derived from the associated manual segmentation. A mean HD of 5.26 ± 0.6 mm was obtained from CMG Net, demonstrating that HD was significantly reduced in our proposed method. After the assembly of all segmented layers according to the original CT sequence, we were able to rebuild an accurately segmented 3D hip model; all anatomical structures and features could be observed clearly (Fig. 3D). Therefore, CMG Net achieved the highest accuracy for the segmentation of normal hips without post-processing.

Furthermore, the mean segmentation time for CMG Net was 23.7 ± 1.0 s on a Nvidia GeForce GTX TITAN X GPU (Table 3), while the mean manual segmentation time was 1612.6 ± 270 s (Table 4). This indicated that the hip joint segmentation times using traditional CNN methods and manual segmentation were approximately 1.5–2.7-fold and 68.0-fold greater than the times for CMG Net.

**Table 3:** Multiple comparison of CMG Net and the order four nets of segmentation time

Disease	(I) NET	(J) NET	Mean difference (I − J)	Std. error	Sig	95% confidence interval	
						Lower bound	Upper bound
Multiple comparisons							
Dependent variable: TIME							
Least significance difference (LSD)							
ONFH	CMG net	FCN	− 10.66	0.36	< 0.05	− 11.37	− 9.95
		2D UNET	− 9.87	0.36	< 0.05	− 10.58	− 9.16
		2.5D UNET	− 31.55	0.36	< 0.05	− 32.26	− 30.84
		3D UNET	− 41.33	0.36	< 0.05	− 42.04	− 40.62
DDH	CMG net	FCN	− 10.22	0.22	< 0.05	− 10.66	− 9.78
		2D UNET	− 10.15	0.22	< 0.05	− 10.59	− 9.71
		2.5D UNET	− 32.52	0.22	< 0.05	− 32.96	− 32.09
		3D UNET	− 40.69	0.22	< 0.05	− 41.13	− 40.25
FNF	CMG net	FCN	− 9.84	0.12	< 0.05	− 10.07	− 9.61
		2D UNET	− 9.81	0.12	< 0.05	− 10.04	− 9.58
		2.5D UNET	− 31.43	0.12	< 0.05	− 31.66	− 31.20
		3D UNET	− 39.93	0.12	< 0.05	− 40.16	− 39.69
OA	CMG net	FCN	− 8.63	0.32	< 0.05	− 9.25	− 8.00
		2D UNET	− 10.51	0.32	< 0.05	− 11.13	− 9.88
		2.5D UNET	− 29.66	0.32	< 0.05	− 30.29	− 29.04
		3D UNET	− 40.06	0.32	< 0.05	− 40.69	− 39.44
NORMAL	CMG net	FCN	− 8.97	0.32	< 0.05	− 9.59	− 8.34
		2D UNET	− 10.83	0.32	< 0.05	− 11.46	− 10.21
		2.5D UNET	− 30.33	0.32	< 0.05	− 30.96	− 29.70
		3D UNET	− 40.23	0.32	< 0.05	− 40.86	− 39.60

**Table 4:** The comparison of segmentation time of CMG Net group and manual group

	NORMAL		DDH		FNF		ONFH		OA	
	Sig. (2-tailed)	Mean difference	Sig. (2-tailed)	Mean difference	Sig. (2-tailed)	Mean difference	Sig. (2-tailed)	Mean difference	Sig. (2-tailed)	Mean difference
Manual	< 0.05	1612.5	< 0.05	6057.6	< 0.05	1832.3	< 0.05	5793.7	< 0.05	6211.9
CMG net	< 0.05	24.3	< 0.05	24.3	< 0.05	24.9	< 0.05	23.4	< 0.05	24.4

### CMG Net ensured the overall accuracy of segmented femur head

**Fig. 4:**
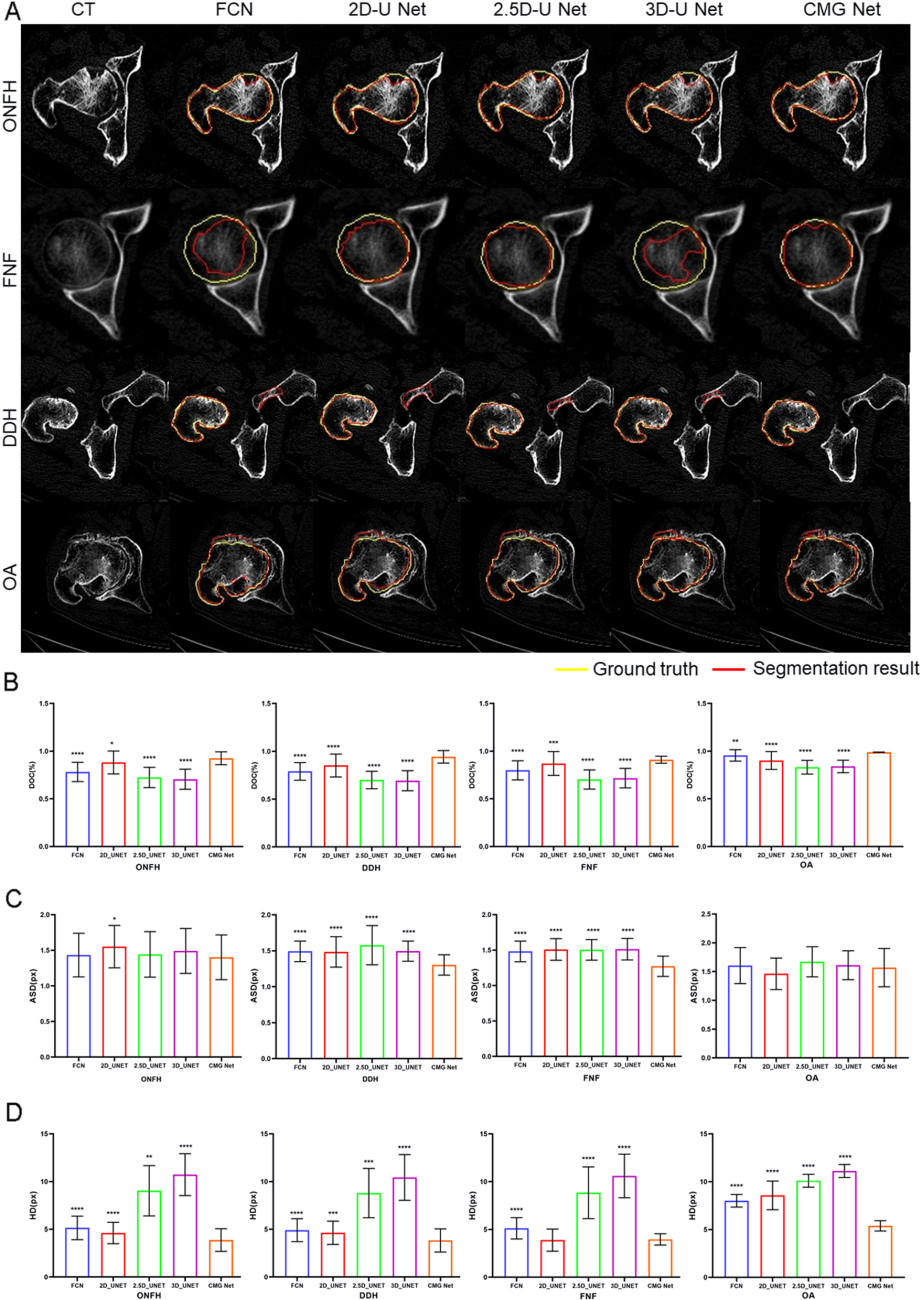
CMG network ensures the overall accuracy of segmented femur head. **A** Qualitative comparison of the segmentation results on a given axial slice of the diseased hip joints (*ONFH* necrosis of the femoral head, *FNF* femoral neck fracture, *DDH* development dysplasia hip, *OA* hip osteoarthritis). **B** Accuracy (DOC, %) comparison between the proposed method and four state-of-the-art methods on diseased hip joints. **C** Accuracy (ASD, px) comparison between the proposed method and four state-of-the-art methods on diseased hip joints. **D** Accuracy (HD, px) comparison between the proposed method and four state-of-the-art methods on diseased hip joints. **p < 0.01, ***p < 0.001, ****p < 0.0001 versus CMG Net

Figure 4A shows the axial views of typical cases. CMG Net achieved acceptable results for the segmentation of diseased hip joints. A comparison among methods in terms of DOC, ASD, and HD is shown in Fig. 4B–D and Table 5. As expected, in the diseased hip segmentation task, CMG Net achieved a DOC of 93.55% ± 5.57%, ASD of 1.34 ± 0.24 mm, and HD of 4.19 ± 1.04 mm. Thus, CMG Net could significantly improve the performance of CNN-based medical image segmentation.

**Table 5:** Multiple comparison of accuracy between CMG Net and the other four nets

Disease	Dependent variable	(I) NET	(J) NET	Mean difference (I − J)	Std. error	Sig	95% confidence interval	
							Lower bound	Upper bound
Multiple comparisons								
Least significance difference (LSD)								
ONFH	DOC	CMG net	FCN	0.14	0.02	< 0.05	0.10	0.19
			2D UNET	0.04	0.02	< 0.05	0.00	0.09
			2.5D UNET	0.20	0.02	< 0.05	0.16	0.24
			3D UNET	0.22	0.02	< 0.05	0.18	0.26
	ASD	CMG net	FCN	− 0.03	0.06	0.61	− 0.15	0.09
			2D UNET	− 0.15	0.06	< 0.05	− 0.27	− 0.03
			2.5D UNET	− 0.04	0.06	0.51	− 0.16	0.08
			3D UNET	− 0.09	0.06	0.14	− 0.21	0.03
	HD	CMG net	FCN	− 1.28	0.36	< 0.05	− 1.99	− 0.57
			2D UNET	− 0.74	0.36	< 0.05	− 1.45	− 0.03
			2.5D UNET	− 5.16	0.36	< 0.05	− 5.87	− 4.45
			3D UNET	− 6.86	0.36	< 0.05	− 7.56	− 6.15
DDH	DOC	CMG net	FCN	0.15	0.02	< 0.05	0.12	0.18
			2D UNET	0.09	0.02	< 0.05	0.06	0.12
			2.5D UNET	0.24	0.02	< 0.05	0.21	0.27
			3D UNET	0.25	0.02	< 0.05	0.22	0.28
	ASD	CMG net	FCN	− 0.19	0.03	< 0.05	− 0.25	− 0.13
			2D UNET	− 0.18	0.03	< 0.05	− 0.24	− 0.12
			2.5D UNET	− 0.27	0.03	< 0.05	− 0.34	− 0.21
			3D UNET	− 0.19	0.03	< 0.05	− 0.26	− 0.13
	HD	CMG net	FCN	− 1.08	0.31	< 0.05	− 1.69	− 0.47
			2D UNET	− 0.80	0.31	< 0.05	− 1.41	− 0.20
			2.5D UNET	− 4.97	0.31	< 0.05	− 5.57	− 4.36
			3D UNET	− 6.60	0.31	< 0.05	− 7.21	− 6.00
FNF	DOC	CMG net	FCN	0.11	0.01	< 0.05	0.09	0.14
			2D UNET	0.04	0.01	< 0.05	0.01	0.06
			2.5D UNET	0.21	0.01	< 0.05	0.18	0.23
			3D UNET	0.19	0.01	< 0.05	0.17	0.22
	ASD	CMG net	FCN	− 0.21	0.02	< 0.05	− 0.25	− 0.17
			2D UNET	− 0.24	0.02	< 0.05	− 0.27	− 0.20
			2.5D UNET	− 0.23	0.02	< 0.05	− 0.27	− 0.19
			3D UNET	− 0.24	0.02	< 0.05	− 0.28	− 0.20
	HD	CMG net	FCN	− 1.16	0.22	< 0.05	− 1.60	− 0.73
			2D UNET	0.08	0.22	0.73	− 0.36	0.51
			2.5D UNET	− 4.88	0.22	< 0.05	− 5.32	− 4.45
			3D UNET	− 6.64	0.22	< 0.05	− 7.08	− 6.21
OA	DOC	CMG net	FCN	0.03	0.02	< 0.05	0.00	0.07
			2D UNET	0.09	0.02	< 0.05	0.05	0.12
			2.5D UNET	0.16	0.02	< 0.05	0.12	0.19
			3D UNET	0.15	0.02	< 0.05	0.12	0.18
	ASD	CMG net	FCN	− 0.04	0.07	0.62	− 0.18	0.11
			2D UNET	0.11	0.07	0.14	− 0.04	0.25
			2.5D UNET	− 0.10	0.07	0.16	− 0.25	0.04
			3D UNET	− 0.04	0.07	0.57	− 0.19	0.10
	HD	CMG net	FCN	− 2.63	0.22	< 0.05	− 3.07	− 2.18
			2D UNET	− 3.19	0.22	< 0.05	− 3.63	− 2.74
			2.5D UNET	− 4.72	0.22	< 0.05	− 5.17	− 4.28
			3D UNET	− 5.74	0.22	< 0.05	− 6.18	− 5.29
NORMAL	DOC	CMG net	FCN	0.01	0.02	0.53	− 0.02	0.04
			2D UNET	0.09	0.02	< 0.05	0.06	0.12
			2.5D UNET	0.15	0.02	< 0.05	0.11	0.18
			3D UNET	0.16	0.02	< 0.05	0.13	0.20
	ASD	CMG net	FCN	− 0.05	0.07	0.53	− 0.19	0.10
			2D UNET	− 0.02	0.07	0.81	− 0.16	0.13
			2.5D UNET	− 0.09	0.07	0.24	− 0.23	0.06
			3D UNET	0.04	0.07	0.60	− 0.11	0.19
	HD	CMG net	FCN	− 2.75	0.27	< 0.05	− 3.28	− 2.22
			2D UNET	− 3.44	0.27	< 0.05	− 3.98	− 2.91
			2.5D UNET	− 4.61	0.27	< 0.05	− 5.14	− 4.08
			3D UNET	− 6.00	0.27	< 0.05	− 6.54	− 5.47

**Fig. 5:**
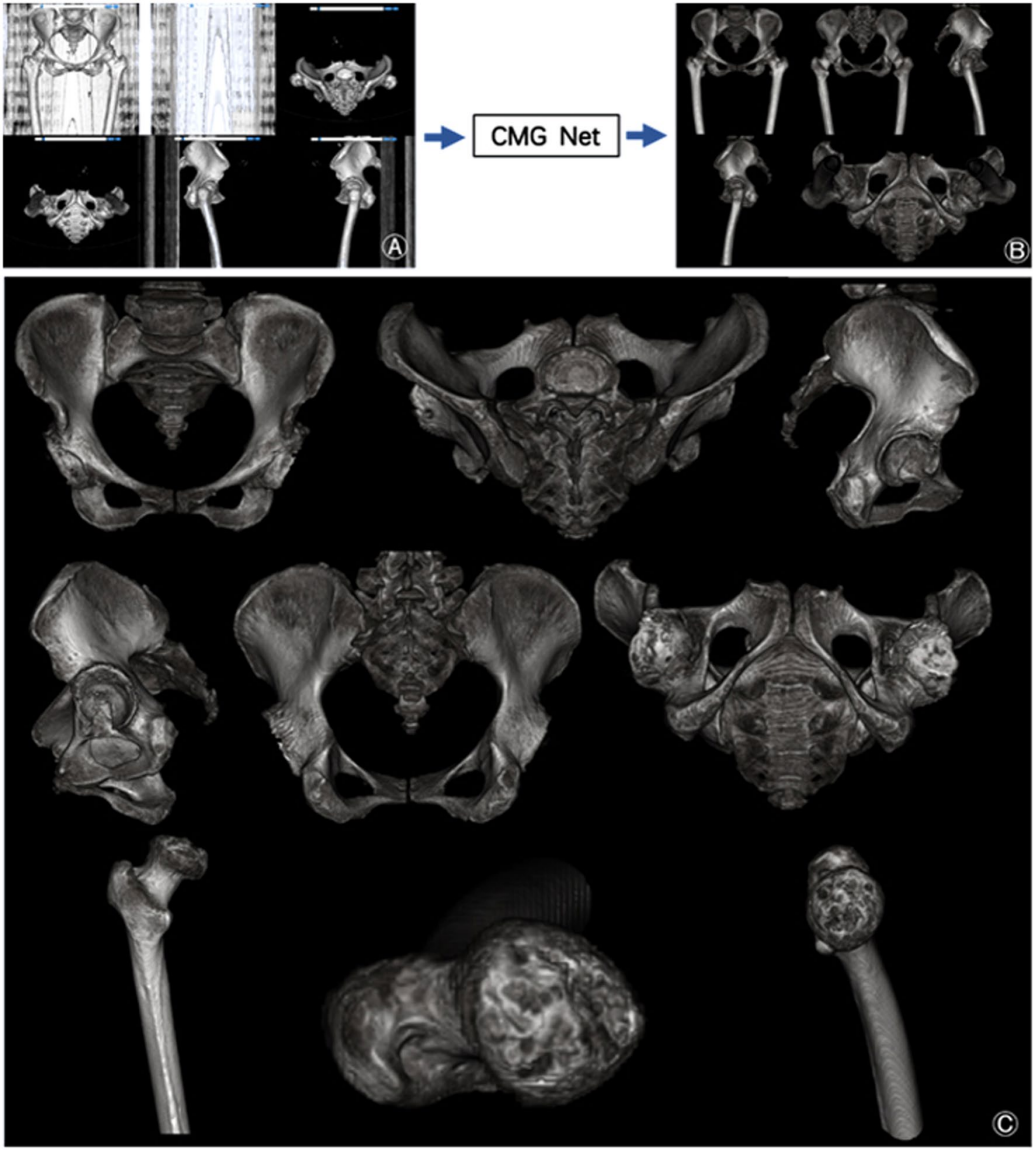
The CMG Net can accurately segment diseased hips. **A** 3D model rebuilt by original CT images; **B** 3D model rebuilt by CT images segmented by CMG Net; **C** the osteophytes and defects of both femur and acetabular can be observed clearly

Paired *t* tests and multiple comparisons in the general linear model showed that DOC, ASD, and HD were significantly better when using CMG Net than when using other methods for both diseased and normal hip segmentation. In subgroup analysis, CMG Net performed better in severe cases, including Crowe III/IV DDH and ARCO stage III/IV ONFH and Garden III/IV FNF (Table 6). After assembly of all segmented layers according to the original CT sequence, all osteophytes and defects could be observed clearly (Fig. 5).

**Table 6:** Subgroup analysis of accuracy between CMGsNET and other four nets in segmentation of severe diseases

Disease	Dependent variable	(I) NET	(J) NET	Mean difference (I − J)	Std. error	Sig	95% confidence interval	
							Lower bound	Upper bound
Multiple comparisons								
Least significance difference (LSD)								
ONFH III	ASD	CMG net	FCN	− 0.19	0.04	< 0.05	− 0.27	− 0.12
			2D UNET	− 0.27	0.04	< 0.05	− 0.34	− 0.19
			2.5D UNET	− 0.25	0.04	< 0.05	− 0.33	− 0.17
			3D UNET	− 0.22	0.04	< 0.05	− 0.30	− 0.14
	DOC	CMG net	FCN	0.10	0.03	< 0.05	0.04	0.15
			2D UNET	0.04	0.03	0.12	− 0.01	0.10
			2.5D UNET	0.20	0.03	< 0.05	0.15	0.25
			3D UNET	0.21	0.03	< 0.05	0.16	0.26
	HD	CMG net	FCN	− 1.10	0.46	< 0.05	− 2.01	− 0.19
			2D UNET	− 0.33	0.46	0.47	− 1.24	0.58
			2.5D UNET	− 5.67	0.46	< 0.05	− 6.58	− 4.76
			3D UNET	− 6.67	0.46	< 0.05	− 7.58	− 5.76
ONFH IV	ASD	CMG net	FCN	0.00	0.07	0.99	− 0.14	0.14
			2D UNET	− 0.09	0.07	0.20	− 0.23	0.05
			2.5D UNET	− 0.09	0.07	0.19	− 0.23	0.04
			3D UNET	− 0.10	0.07	0.15	− 0.24	0.04
	DOC	CMG net	FCN	0.14	0.03	< 0.05	0.09	0.19
			2D UNET	0.05	0.03	0.05	0.00	0.10
			2.5D UNET	0.21	0.03	< 0.05	0.16	0.26
			3D UNET	0.23	0.03	< 0.05	0.18	0.28
	HD	CMG net	FCN	− 1.31	0.40	< 0.05	− 2.11	− 0.52
			2D UNET	− 0.77	0.40	0.06	− 1.57	0.02
			2.5D UNET	− 5.10	0.40	< 0.05	− 5.89	− 4.30
			3D UNET	− 7.13	0.40	< 0.05	− 7.92	− 6.34
DDH III	ASD	CMG net	FCN	− 0.12	0.05	< 0.05	− 0.23	− 0.02
			2D UNET	− 0.18	0.05	< 0.05	− 0.28	− 0.07
			2.5D UNET	− 0.37	0.05	< 0.05	− 0.47	− 0.26
			3D UNET	− 0.13	0.05	< 0.05	− 0.24	− 0.03
	DOC	CMG net	FCN	0.18	0.03	< 0.05	0.11	0.24
			2D UNET	0.07	0.03	< 0.05	0.01	0.13
			2.5D UNET	0.22	0.03	< 0.05	0.15	0.28
			3D UNET	0.26	0.03	< 0.05	0.20	0.33
	HD	CMG net	FCN	− 1.46	0.67	< 0.05	− 2.80	− 0.11
			2D UNET	− 0.15	0.67	0.83	− 1.49	1.20
			2.5D UNET	− 5.06	0.67	< 0.05	− 6.41	− 3.72
			3D UNET	− 6.60	0.67	< 0.05	− 7.94	− 5.25
DDH IV	ASD	CMG net	FCN	− 0.21	0.07	< 0.05	− 0.34	− 0.08
			2D UNET	− 0.20	0.07	< 0.05	− 0.34	− 0.07
			2.5D UNET	− 0.25	0.07	< 0.05	− 0.38	− 0.12
			3D UNET	− 0.17	0.07	< 0.05	− 0.30	− 0.04
	DOC	CMG net	FCN	0.13	0.04	< 0.05	0.05	0.20
			2D UNET	0.12	0.04	< 0.05	0.05	0.19
			2.5D UNET	0.28	0.04	< 0.05	0.21	0.35
			3D UNET	0.28	0.04	< 0.05	0.20	0.35
	HD	CMG net	FCN	− 1.29	0.67	0.06	− 2.64	0.07
			2D UNET	− 1.50	0.67	< 0.05	− 2.85	− 0.14
			2.5D UNET	− 5.47	0.67	< 0.05	− 6.83	− 4.12
			3D UNET	− 6.39	0.67	< 0.05	− 7.74	− 5.03
FHF III	ASD	CMG net	FCN	− 0.21	0.02	< 0.05	− 0.26	− 0.16
			2D UNET	− 0.24	0.02	< 0.05	− 0.29	− 0.20
			2.5D UNET	− 0.24	0.02	< 0.05	− 0.28	− 0.19
			3D UNET	− 0.24	0.02	< 0.05	− 0.29	− 0.19
	DOC	CMG net	FCN	0.11	0.02	< 0.05	0.08	0.14
			2D UNET	0.04	0.02	< 0.05	0.01	0.08
			2.5D UNET	0.20	0.02	< 0.05	0.17	0.23
			3D UNET	0.18	0.02	< 0.05	0.15	0.21
	HD	CMG net	FCN	− 1.21	0.28	< 0.05	− 1.76	− 0.67
			2D UNET	0.21	0.28	0.45	− 0.34	0.75
			2.5D UNET	− 4.53	0.28	< 0.05	− 5.08	− 3.99
			3D UNET	− 6.38	0.28	< 0.05	− 6.93	− 5.84
FHF IV	ASD	CMG net	FCN	− 0.19	0.04	< 0.05	− 0.27	− 0.12
			2D UNET	− 0.27	0.04	< 0.05	− 0.34	− 0.19
			2.5D UNET	− 0.25	0.04	< 0.05	− 0.33	− 0.17
			3D UNET	− 0.22	0.04	< 0.05	− 0.30	− 0.14
	DOC	CMG net	FCN	0.10	0.03	< 0.05	0.04	0.15
			2D UNET	0.04	0.03	< 0.05	− 0.01	0.10
			2.5D UNET	0.20	0.03	< 0.05	0.15	0.25
			3D UNET	0.21	0.03	< 0.05	0.16	0.26
	HD	CMG net	FCN	− 1.10	0.46	< 0.05	− 2.01	− 0.19
			2D UNET	− 0.33	0.46	0.47	− 1.24	0.58
			2.5D UNET	− 5.67	0.46	< 0.05	− 6.58	− 4.76
			3D UNET	− 6.67	0.46	< 0.05	− 7.58	− 5.76

## Discussion

In this study, we found that the novel CNN-CMG Net could effectively and accurately segment hip joints from CT images. To our knowledge, this study presents one of the first examples of deep CNN for the automatic segmentation of CT images of normal and diseased hip joints.

A computer-aided segmentation strategy depends on the segmentation accuracy at the edges between the femoral head and acetabulum. However, the boundaries are often ambiguous; the images are frequently affected by degradation, noise, and non-homogeneous intensities in diseased cases. Therefore, we combined the advantages of U-Net and Dense-Net to accelerate the learning process and improve the accuracy of segmentation involving diseased hip joints, while using fewer parameters. CMG net considers bone separation, as well as the features of the edge between bone and soft tissue. We aimed to separate femur head and acetabulum with a high accuracy; thus, we divided the target into two sub-tasks. First, we separated femur head and acetabulum, both of which are bony structures. Second, we maintained the accuracy of the femur head during the bone structure separation process. The bone-soft tissue boundary is critical for the second sub-task. The CMG network utilises two parallel networks to share the responsibility of the two sub-tasks mentioned above. The upper module provides the feature map of the bone-soft tissue interface and fuses to the main separation network to ensure overall accuracy regarding the segmented femur head.

Our proposed strategies for managing diseased hips greatly increased the segmentation accuracy and reduced the mean standard deviation. Compared to traditional CNN nets and manual segmentation [18], the segmentation time of the diseased hip joints using our proposed method was significantly reduced. Shinichi et al. [4] showed a coarse-to-fine hip CT segmentation framework that consisted of regional growth-based preprocessing, conditional random field-based coarse segmentation, and patch-based refinement. Radiology experts expend considerable effort in completing the training samples. Gwun Jang et al. [9] proposed a fully automated segmentation method for hip joints using the complementary characteristics of patient-specific optimal thresholding and the watershed algorithm. However, the use of primitive spheres in the proposed method may be ineffective for CT data in cases where the femoral head is severely deformed because of diseases (e.g. avascular necrosis). Our results suggest that CMG Net is a practical and useful instrument for the segmentation of diseased hips, as well as the observation of all anatomical features. The results also suggest that our proposed strategy was highly practical and clinically useful because it rapidly achieved fully automated and accurate segmentation.

There were several limitations in the present study. First, the overall design of the network was traditional and lacked breakthrough innovations with respect to feature extraction. Second, the task setting was simple, only involving segmentation of the bony parts; it did not include further diagnosis or scoring. Third, the use of this technique was limited to patients without metal implants because metal artefacts could influence the segmentation process. We plan to investigate these issues in subsequent research.

## Conclusion

We present a fully automatic and accurate deep neural network, CMG Net, which is more efficient than existing networks. It achieved a segmentation accuracy comparable to human experts with a shorter run-time. Therefore, CMG Net is highly practical and clinically useful; it may be extended to the segmentation of CT data involving other anatomical structures.

## Data Availability

The datasets are available from the corresponding authors on reasonable request.
